# Amyloid-β Peptide Impact on Synaptic Function and Neuroepigenetic Gene Control Reveal New Therapeutic Strategies for Alzheimer’s Disease

**DOI:** 10.3389/fnmol.2020.577622

**Published:** 2020-11-13

**Authors:** Bhanu Chandra Karisetty, Akanksha Bhatnagar, Ellen M. Armour, Mariah Beaver, Haolin Zhang, Felice Elefant

**Affiliations:** Department of Biology, Drexel University, Philadelphia, PA, United States

**Keywords:** neuroepigenetics, TIP60, synaptic function, therapeutics, amyloid beta, KAT5

## Abstract

Amyloid-β (Aβ) peptides can form protease-resistant aggregates within and outside of neurons. Accumulation of these aggregates is a hallmark of Alzheimer’s disease (AD) neuropathology and contributes to devastating cognitive deficits associated with this disorder. The primary etiological factor for Aβ aggregation is either an increase in Aβ production or a decrease in its clearance. Aβ is produced by the sequential activity of β- and γ-secretase on the amyloid precursor protein (APP) and the clearance is mediated by chaperone-mediated mechanisms. The Aβ aggregates vary from soluble monomers and oligomers to insoluble senile plaques. While excess intraneuronal oligomers can transduce neurotoxic signals into neurons causing cellular defects like oxidative stress and neuroepigenetic mediated transcriptional dysregulation, extracellular senile plaques cause neurodegeneration by impairing neural membrane permeabilization and cell signaling pathways. Paradoxically, senile plaque formation is hypothesized to be an adaptive mechanism to sequester excess toxic soluble oligomers while leaving native functional Aβ levels intact. This hypothesis is strengthened by the absence of positive outcomes and side effects from immunotherapy clinical trials aimed at complete Aβ clearance, and support beneficial physiological roles for native Aβ in cellular function. Aβ has been shown to modulate synaptic transmission, consolidate memory, and protect against excitotoxicity. We discuss the current understanding of beneficial and detrimental roles for Aβ in synaptic function and epigenetic gene control and the future promising prospects of early therapeutic interventions aimed at mediating Aβ induced neuroepigenetic and synaptic dysfunctions to delay AD onset.

## Introduction

Alzheimer’s disease (AD) affects 5.8 million Americans aged 65 and older and is estimated to grow to 13.8 million by mid-century. AD is the most common cause of dementia, presenting with hallmarks such as amyloid-β (Aβ) plaques, tau neurofibrillary tangles, neuronal cell death, cognitive dysfunction, and altered brain morphology. Aβ-plaques comprise of aggregated Aβ, a cleaved product of the glycoprotein amyloid precursor protein (APP). According to the amyloid cascade hypothesis, it is these plaques that are responsible for AD pathology.

Newly generated Aβ released into the extracellular space remain in soluble form or aggregate into insoluble Aβ-plaques. Soluble Aβ species can bind to various neuronal cell receptors and transduce neurotoxic signals causing cellular defects that include oxidative stress and epigenetic-mediated transcriptional dysregulation (Chen et al., 2017). However, recent evidence demonstrates that soluble Aβ shows beneficial physiological roles such as regulating cellular signaling pathways and synaptic function as well as possessing antimicrobial and antioxidant properties (Brothers et al., 2018). In this review, we summarize recent progress in understanding the beneficial and detrimental aspects of Aβ in mediating processes underlying synaptic and cognitive function and epigenetic neuronal gene control. We further discuss therapeutic interventions aimed at synaptic plasticity and epigenetic regulation to delay AD progression.

## Aβ Regulation of Synaptic Plasticity

Synaptic plasticity mediated changes in neuronal connections have long been established as the primary mechanism of learning and memory (Martin et al., 2000; Ramirez and Arbuckle, 2016). Accordingly, loss of synaptic connections is an early event in AD pathogenesis and cognitive impairment (Selkoe, 2002; Scheff et al., 2006; Kashyap et al., 2019). Although the precise mechanisms underlying synaptic dysfunction in AD are obscure, emerging studies have uncovered a feedback regulation between Aβ and synaptic plasticity.

**FIGURE 1:**
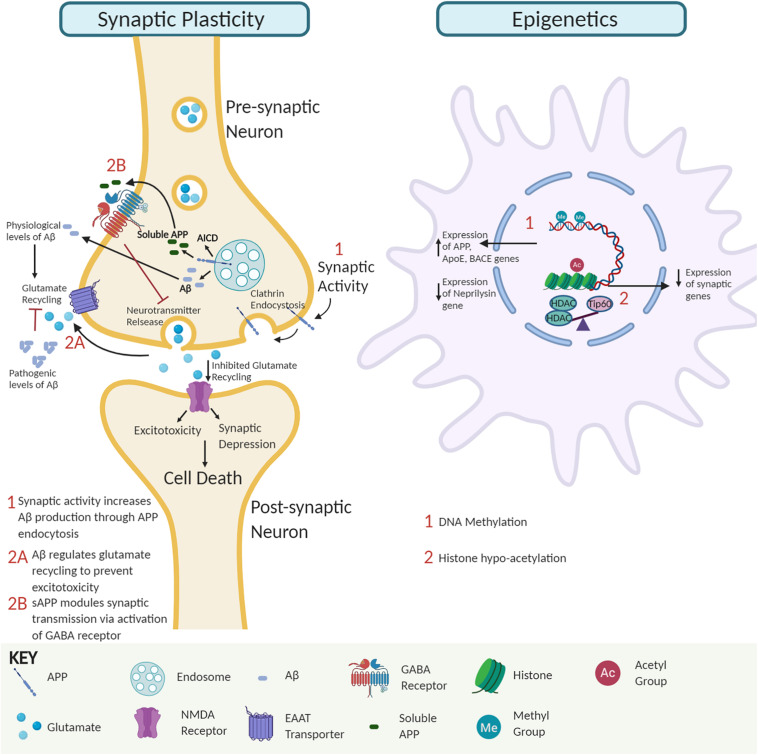
The interplay of amyloid precursor protein (APP) and amyloid-β (Aβ) impact on synaptic activity and neuroepigenetic gene control. Synaptic activity module. APP endocytosis and cleavage yield Aβ peptides and APP intracellular domain (AICD). Soluble APP regulates GABA receptor function to modulate synaptic transmission and plasticity. At physiological levels, Aβ peptides promote glutamate recycling keeping glutamate levels in check. In contrast, at pathological Aβ levels, impaired glutamate recycling increases post-synaptic uptake, leading to excitotoxicity, synaptic depression, and eventually neural cell death. Epigenetic module. Soluble Aβ monomer and dimer competition for binding of essential ligands to receptors can alter signal transduction pathways causing global changes in neuroepigenetic gene control. Such alterations include DNA methylation changes that alter the expression of genes involved in APP processing and Aβ degradation pathways to favor increased Aβ levels and disruption of Tip60/HDAC balance causing hypoacetylation of chromatin with concomitant repression of associated critical synaptic plasticity genes.

Multiple studies demonstrate that soluble Aβ oligomers in pre- and post-synaptic compartments can disrupt synaptic morphology and inhibit long-term potentiation (LTP) that trigger cognitive dysfunction. Intriguingly, insoluble Aβ-plaques are less active in promoting such alterations (Lambert et al., 1998; Takahashi et al., 2002; Walsh et al., 2002; Shankar et al., 2008; Figure 1). For example, studies in amyloid mice reveal that reduction of synaptophysin puncta correlates with soluble Aβ and not plaque load (Mucke et al., 2000). Further, AD-associated apolipoprotein E4 has been implicated in facilitating the transport of soluble Aβ species to synapses elucidating toxic effects (Koffie et al., 2012). Aberrant activation of neuronal signal transduction pathways can arise via Aβ directly binding to Aβ receptors or competing with essential ligands to bind their receptors (Xia et al., 2016). For example, soluble Aβ dimers cause glutamate excitotoxicity via blockage of glutamate reuptake in the synaptic cleft, activating glutamate receptors and ion channels like N-Methyl-D-aspartate (NMDA) receptors that trigger downstream cell signaling transduction cascades to pathologically alter gene expression profiles (Li et al., 2009). Additionally, accumulation of extracellular Aβ_42_ triggers the loss of synaptic mushroom spines via hyperactivation of metabotropic glutamate receptor type 5 (mGluR5) receptors, resulting in elevated endoplasmic reticulum Ca^2+^ levels and downregulation of the Ca^2+^/calmodulin kinase II signaling pathway (Zhang et al., 2015). Interestingly, soluble APP has also been shown to directly modulate synaptic plasticity by binding to the gamma-aminobutyric acid (GABA) receptor and inducing a conformational change that facilitates reduced neurotransmitter release and neuronal activity (Rice et al., 2019; Figure 1). Together, these findings support the concept that soluble APP and Aβ oligomers promote synaptic impairment and cognitive deficits during the early stages of AD, followed by neurodegeneration in the later stages (Ferreira and Klein, 2011).

Conversely, synaptic activity positively modulates Aβ production to promote synaptic function (Kamenetz et al., 2003; Cirrito et al., 2005). Increased synaptic activity promotes APP endocytosis, and β-secretase 1 (BACE1) mediated Aβ production (Cirrito et al., 2008). Since Aβ depresses synaptic activity, the activity-dependent modulation of endogenous Aβ production has been suggested to be a finely tuned negative feedback loop that regulates the neuronal activity and appropriate function by preventing hyperactivation (Kamenetz et al., 2003). Perturbation in this homeostatic mechanism may interfere with synaptic activity and contribute to cognitive decline, as seen in AD. These studies support the premise that physiological levels of Aβ are critical for optimal synaptic activity (Kamenetz et al., 2003; Parihar and Brewer, 2010; Jang and Chung, 2016).

## Aβ and Epigenetic Mechanisms Underlying AD

Epigenetic modifications of DNA and histone proteins regulate gene expression profiles via controlling chromatin accessibility. The neuroepigenome has been proven to be critical in memory formation and consolidation through dynamic control of neural genes essential for these functions (Feng et al., 2007; Sultan and Day, 2011). Neuroepigenetic imbalance in the brain causes transcriptional dysregulation, a pivotal step in AD etiology (Esposito and Sherr, 2019). Here, we summarize primary epigenetic alterations that affect or are affected by Aβ production.

**DNA methylation:** DNA methylation occurs at cytosine bases in CpG repeats and primarily controls gene repression (Saxonov et al., 2006; Miranda and Jones, 2007). Reports on DNA methylation and AD are conflicting with several studies reporting global DNA hypermethylation in the AD brain (Rao et al., 2012; Di Francesco et al., 2015; Liu et al., 2019), while other studies show reduction (Chen et al., 2009; Chouliaras et al., 2013; Li et al., 2019) or no alterations in global DNA methylation (Lashley et al., 2015). Common AD-associated methylation alterations often increase Aβ production. For example, AD-associated genes APP, Apolipoprotein E, and BACE1 are hypomethylated in AD brains with concomitant BACE1 activation increasing Aβ levels via the amyloidogenic processing pathway (West et al., 1995; Tulloch et al., 2018; Li et al., 2019). Conversely, the neprilysin gene that encodes for an Aβ degrading enzyme is hypermethylated and repressed in AD, also leading to increased Aβ levels (Chen et al., 2009).

**Histone acetylation:** Histone modifications, including acetylation, methylation, and phosphorylation on histone protein tails, modulate chromatin accessibility to control gene expression. Of these modifications, histone acetylation is best characterized for its role in learning and memory and contribution to AD when altered (Saha and Pahan, 2006; Sharma, 2010; Peixoto and Abel, 2013). Histone acetylation homeostasis is regulated by the antagonistic activity of histone acetyltransferases (HATs) and histone deacetylases (HDACs). Evidence from our group and others shows that neural histone acetylation dysregulation, caused by an imbalance between specific HATs and HDACs, is a crucial early step in AD pathology. Downregulation of the HAT Tip60 (KAT5) and upregulation of HDAC2 causes epigenetic repression of critical neuroplasticity genes in multiple types of AD animal models and patients (Graff et al., 2012; Panikker et al., 2018). Further, alteration of Tip60 epigenetic mediated control in the brain by either APP or Aβ driven Alzheimer’s disease pathology leads to repression of a set of neuronal genes critical for synaptic function (Panikker et al., 2018). Restoring such alterations in Tip60/HDAC2 balance protects against AD-associated pathologies in the AD *Drosophila* model expressing APP.

How might Aβ influence early HAT/HDAC disruption? Our findings reveal that APP expression results in a reduction of Tip60 protein levels but not Tip60 mRNA levels (Panikker et al., 2018), suggesting a mechanism of post-transcriptional regulation. Thus far, there is no evidence to demonstrate a direct Aβ and Tip60 interaction underlying reduced Tip60 levels, but a potential mechanism is via ubiquitin-mediated Aβ-induced Tip60 degradation. Another consideration is soluble Aβ monomer and dimer competition for the binding of essential ligands to receptors (Xia et al., 2016). Such interactions possibly alter signal transduction pathways that disrupt Tip60/HDAC balance and acetylation levels, inducing altered gene expression profiles contributing to AD.

## Therapeutic Intervention for Aβ Induced Neuroepigenetic and Synaptic Dysfunction

Currently, the Food and Drug Administration (FDA) approved drugs for AD are limited to palliative medications: cholinesterase inhibitors and a non-competitive NMDA antagonist (National Institute on Aging, 2018, April 01). As Aβ-plaques are considered as primary effector molecules in AD pathogenesis, therapeutic strategies are focused on developing agents that can block Aβ production or clear Aβ-plaques. The clinical trials are ongoing, but the initial results thus far are not encouraging. The β- and γ-secretase inhibitors, aimed to block Aβ production, were discontinued due to unfavorable risk/benefit profile and cognitive worsening. Also, Aβ immunotherapies, intended to clear the Aβ-plaques, were terminated due to toxicity and cognitive worsening. Efforts are in progress to refine the approaches to these trials (reviewed in Panza et al., 2019). Further, it is hypothesized that the complete reduction of Aβ as a principal reason for these failures, underscoring the necessity to understand the physiological roles of Aβ.

The U-shaped natural course of cerebrospinal fluid Aβ levels in aging suggests it as physiologically active (Shoji and Kanai, 2001). One of the main reasons for clinical trial failures is the toxicity resulting from reducing Aβ, supporting a critical role for Aβ in neuronal survival and function. In support of this concept, synthetic Aβ_42_ monomers (30–100 nM) have been shown to promote survival in developing neurons deprived with trophic factors (Giuffrida et al., 2009). Further, in different neuronal cell types, exogenous Aβ_40_ had a neuroprotective effect on cells dying from Aβ immunodepletion, while the same levels of exogenous Aβ_42_ oligomers proved to be toxic (Plant et al., 2003). These studies demonstrate a hormetic effect of Aβ in neuroprotection and the neurotoxicity of soluble oligomeric forms over insoluble aggregates. Considering the physiological importance of monomeric Aβ, monoclonal antibody Aducanumab was developed with a much greater affinity to Aβ-aggregates versus monomeric forms. Currently, Phase 3 trials have been discontinued based on futility analysis but not on safety concerns (U.S. National Library of Medicine, 2020a,b). In the future, the predicted aggregate-specific N-terminal binding motif of Aducanumab could potentially serve as a basis to re-engineer Aducanumab for further enhanced preference to bind Aβ -aggregates versus monomers (Frost and Zacharias, 2020).

Another disappointing outcome from clinical trials focused on Aβ depletion is the failure to alleviate cognitive decline. Studies show that Aβ affects memory by regulating synaptic vesicle dynamics and synaptic plasticity with physiological levels increasing recycling and supraphysiological levels decreasing recycling (Lazarevic et al., 2017). Similarly, exogenously applied Aβ_42_ shows a biphasic dose-response curve on hippocampal LTP and reference memory (Puzzo et al., 2012). Additional studies carried out to understand the synaptic plasticity and memory formation by different isoforms (Aβ_40_ and Aβ_42_) and aggregation status (monomer and oligomer) revealed that lower levels of oligomeric Aβ_42_ enhanced LTP and spatial memory while higher concentrations of oligomeric Aβ_40_, oligomeric Aβ_42_ & monomeric Aβ_42_ impaired LTP and spatial memory (Gulisano et al., 2018). In addition to memory formation, Aβ is required for memory consolidation and stability. Intrahippocampal administration of picomolar concentrations of exogenous Aβ_42_, following training, enhances memory retention (Garcia-Osta and Alberini, 2009). Elevated soluble Aβ_42_ in the amygdala of adult rats, during the formation of auditory fear memories, is required for memory consolidation and stability (Finnie and Nader, 2020). These studies signify the importance of physiological concentrations of Aβ on memory formation and retention and substantiate the hypothesis that cognitive deficits increase due to Aβ depletion.

**FIGURE 2:**
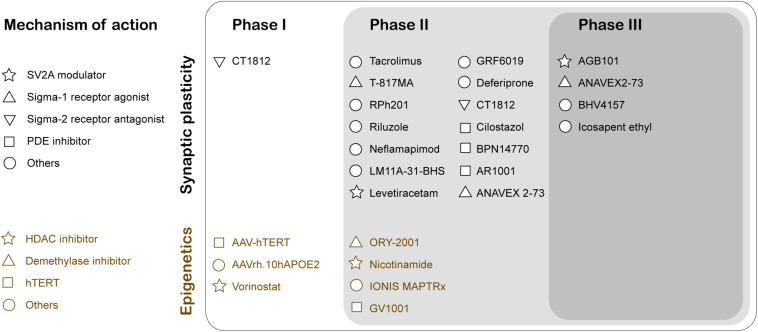
Clinical Trials for therapeutic drugs targeting synaptic activity and neuroepigenetic mechanisms for Alzheimer’s disease treatment. The mechanism of action of the drug is classified using Common Alzheimer’s and Related Dementias Research Ontology (CADRO) (Cummings et al., 2020). SV2A, Synaptic vesicle glycoprotein 2A; PDE, Phosphodiesterase; HDAC, Histone deacetylases; hTERT, human telomerase reverse transcriptase.

The two major histopathological hallmarks of AD are extracellular Aβ-plaques and intracellular neurofibrillary tangles. These changes predominantly occur in the later stages of AD. In contrast, synaptic dysfunction typically appears early in prodromal or mild cognitive impairment (MCI) stages of the disease, thus serving as a potent target for early stage therapeutic intervention to slow AD progression. Soluble Aβ_42_ oligomers can interact with proteins participating in the regulation of the synaptic vesicle life cycle that includes Syntaxin1a, Synaptophysin, and Synapsin1 (Snp1), causing aberrant glutamate release and reduction in synaptic vesicle recycling (reviewed in Marsh and Alifragis, 2018). Currently, there are three publicly disclosed drug trials with endpoints that specifically inform on synapse density and/or function (reviewed in Jackson et al., 2019). First, Elayta (CT1812) is a small-molecule that prevents and displaces beta-amyloid binding to the sigma-2 receptor on the nerve cells and interferes with its toxicity. Elayta lowered the neurogranin and synaptotagmin-1, markers of synaptic damage, in AD patients (U.S. National Library of Medicine, 2020d). A second trial is using imaging techniques and cognitive performance testing to assess the efficiency of LMTX (methylthioninium chloride), a tau aggregation inhibitor, to elicit changes in brain function (U.S. National Library of Medicine, 2020c). Finally, Saracatinib inhibition of Fyn is another potential synaptic specific therapeutic intervention in AD. Fyn is a non-receptor tyrosine kinase that is activated by Aβ oligomers and alters synaptic plasticity (U.S. National Library of Medicine, 2019). These studies have moved the field forward toward clinical trials testing therapuetic drugs designed specifically for synaptic plasticity enhancement (Figure 2). Indeed, recent years have shown an increase in the number of drugs/biologics targeting pathways other than amyloid or tau (Cummings et al., 2020).

Compelling evidence demonstrates that repression of histone acetylation mediated epigenetic gene control involving an increase in HDAC2 and a reduction of Tip60 are early contributors to AD (Panikker et al., 2018). Thus, epigenetic therapeutic approaches that involve increasing acetylation levels using HDAC inhibitors (HDACi) and HAT activators is a promising therapeutic approach. At present, there are two HDACi at clinical trials targeting AD pathology (Figure 2; Cummings et al., 2020). Nicotinamide is at Phase 2 testing to assess the reduction of phosphorylated tau in patients that display MCI or mild AD dementia. Another HDACi vorinostat is in Phase 1b to determine the maximal tolerable dose in AD patients between (including) 55 and 90 years with mild symptoms. The epigenetic drug valproic acid restores the physiological regulation of Snp1, a pre-synaptic protein that regulates the availability of synaptic vesicles, in Aβ_42_ treated primary rat hippocampal neurons (Marsh et al., 2017). Mithramycin A (FDA approved antineoplastic antibiotic) significantly upregulates the synaptic plasticity gene expression and downregulates HDAC2 in SH-SY5Y cells overexpressing APP (Atluri et al., 2019).

HDACi can be either multitargeting like M344, an inhibitor of class I and IIB HDACs, or specific like CM-695 and RGFP966 selectively inhibiting HDAC6 and HDAC3, respectively. M344 regulated multiple AD-related genes and exhibited significant cognitive benefits *in vivo* (Volmar et al., 2017). CM-695 inhibits HDAC6 and phosphodiesterase 9 (PDE9) and ameliorates memory impairment, and reduces Aβ_42_ levels *in vivo* (Cuadrado-Tejedor et al., 2019). RGFP966 inhibits HDAC3 and reverse the attenuation of LTP by Aβ oligomers in rat CA1 pyramidal neurons (Krishna et al., 2016). Selective inhibition by specific HDACi can reduce the side effects and serves as a viable therapeutic strategy. An alternative approach to improve target specificity is to target the binding partners in the HDAC complex rather than the HDAC. Utilizing weighted gene co-expression network analysis (WGCNA), transcription factor Sp3 identified as a putative HDAC2 co-regulator, and its expression was also elevated in AD patients. The knockdown of Sp3 reduced the HDAC2 occupancy and reversed the HDAC2 associated synaptic gene repression (Yamakawa et al., 2017). Therefore, targeting the HDAC2-Sp3 complex may be a feasible approach for AD therapy.

Many HATs as opposed to HDACs have non-redundant physiological functions as different HATs exhibit a specialized preference for site-specific chromatin marks that regulate synaptic gene expression and cognitive function. Thus, HAT activators are a potentially powerful epigenetic therapeutic tool for the treatment of neurodegenerative diseases. As such, chemical modifications are being made to existing drugs to increase their cell permeability in the brain. For example, TTK21, an activator of the HAT CBP/p300, is conjugated to a glucose-based carbon nanosphere enabling it to cross the blood-brain barrier (Chatterjee et al., 2013). It promotes neurogenesis and extends memory duration *in vivo*. A patent publication (US20180050982A1) covers the use of HAT activators to enhance learning and memory and to treat AD (Francis et al., 2018). Alternatively, downstream molecules/pathways regulated by HATs can also be targeted for therapeutic effects.

A recent growing interest among many researchers is moving toward exploration of non-coding RNA (ncRNA) related neuroepigenetic alterations in AD and its relationship with synaptic dysfunction. Notably, some microRNAs (miRNAs) are particularly enriched in presynaptic and postsynaptic compartments. For example, miR-34a can target the synaptic proteins synaptotagmin-1 and syntaxin-1A to regulate neurite outgrowth and dendritic spine morphology and function (Agostini et al., 2011). Further, in AD-associated HDAC2-induced tauopathy, 5′ AMP-activated protein kinase (AMPK) activation is correlated with the loss of spine density. AMPK expression is under the control of the miR-101b promoter and as such, miR-101b mimics have been shown to block dendritic impairments *in vitro* (Liu et al., 2017). Thus, understanding the various ncRNAs in AD pathology should lead to new pharmacological interventions. Interestingly, non-pharmacological approaches like an enriched environment (EE) and non-invasive brain stimulation techniques can be utilized to attenuate early stage synaptic dysfunction and appear to act via neuroepigenetic mechanisms. For example, EE triggers hippocampal induction of histone acetylation at specific sites linked to synaptic plasticity and learning and memory enhancement and also ameliorates soluble Aβ oligomer induced synaptic dysfunction by upregulating miRNA-132 and reducing HDAC3 signaling (Wei et al., 2020). Currently, transcranial magnetic stimulation (TMS) and transcranial direct current stimulation (tDCS) usage have been shown to be beneficial for stroke and Parkinson’s patients to positively modulate brain plasticity (reviewed in Schulz et al., 2013). Thus, exciting new avenues involving these types of non-invasive treatment methods likely hold promise for AD patients as well.

## Outlook

In AD patients, Aβ accumulation and associated neuroepigenetic transcriptional alterations contribute to synaptic dysfunction and cognitive impairment (Panikker et al., 2018). However, failure to attenuate or reverse the cognitive decline by anti-amyloid therapeutics in clinical trials raises concerns toward these strategies. Intriguingly, recent studies demonstrate roles for Aβ in neuroprotection, synaptic function, and memory consolidation (Giuffrida et al., 2009; Lazarevic et al., 2017; Finnie and Nader, 2020). These beneficial roles are Aβ concentration- and species-specific. Picomolar concentrations and monomers proved to be beneficial, while higher concentrations and soluble oligomers proved to be detrimental. These findings underscore the necessity to understand the physiological and pathological roles of Aβ for refining the current amyloid-based therapeutic strategies. As AD is a multifactorial disease, targeting AD-associated processes like tau-associated pathology, inflammatory responses, synaptic activity, and neuroepigenetic regulation of AD-related genes may provide alternative therapeutic strategies during early AD progression. Additionally, exploring the synergistic effects of HDACi and HAT activators to restore histone acetylation homeostasis, opens new less invasive and early avenues for treatment. Recent studies utilizing methodological improvements to specifically target toxic Aβ species demonstrate encouraging results. Thus, the development of early therapeutic interventions aimed at mediating Aβ induced neuroepigenetic and synaptic dysfunctions while simultaneously maintaining beneficial physiological levels and forms of Aβ provide exciting new avenues for preventing or treating AD.

## Author Contributions

BK, AB, EMA, FE, and HZ contributed to writing and reviewing the manuscript. MB and BK contributed to figures and reviewing the manuscript. All authors contributed to the article and approved the submitted version.

## Conflict of Interest

The authors declare that the research was conducted in the absence of any commercial or financial relationships that could be construed as a potential conflict of interest.
